# Severe malaria

**DOI:** 10.1186/s12936-022-04301-8

**Published:** 2022-10-06

**Authors:** Nicholas J. White

**Affiliations:** 1 Mahidol Oxford Research Unit, Faculty of Tropical Medicine, Mahidol University, Bangkok, Thailand; 2 Centre for Tropical Medicine and Global Health, Nuffield Department of Medicine, University of Oxford, Oxford, UK

## Abstract

Severe malaria is a medical emergency. It is a major cause of preventable childhood death in tropical countries. Severe malaria justifies considerable global investment in malaria control and elimination yet, increasingly, international agencies, funders and policy makers are unfamiliar with it, and so it is overlooked. In sub-Saharan Africa, severe malaria is overdiagnosed in clinical practice. Approximately one third of children diagnosed with severe malaria have another condition, usually sepsis, as the cause of their severe illness. But these children have a high mortality, contributing substantially to the number of deaths attributed to ‘severe malaria’. Simple well-established tests, such as examination of the thin blood smear and the full blood count, improve the specificity of diagnosis and provide prognostic information in severe malaria. They should be performed more widely. Early administration of artesunate and broad-spectrum antibiotics to all children with suspected severe malaria would reduce global malaria mortality.

Severe malaria is important. It is a major cause of preventable childhood death in tropical countries. This large number of avoidable deaths justifies the substantial global investments in malaria control and elimination. But severe malaria is increasingly overlooked by the international agencies, donors and policy makers who determine the direction and support for global malaria initiatives.

## History

Severe malaria, or ague, was recognized long before discovery of the malaria parasite by Laveran in 1880. The Cinchona bark arrived in Europe nearly four hundred years ago providing, for the first time, a potential cure for the pervasive and dangerous illness that then affected most of the inhabited world. But, as today, the specificity of the clinical diagnosis of febrile illnesses was poor. Torti recognized that only some fevers could be cured by the bark [1]. Even after the malaria parasite was identified first in 1880, severe forms such as algid malaria (shock), haemorrhagic or gastrointestinal malaria bore an uncertain relationship to *Plasmodium* infection, as did the notorious “blackwater fever”. Until the 1980s, the majority of research on severe malaria was conducted in adults. It derived largely from war-time experiences in the military, or observations from colonial medical services. Specific anti-malarial treatment comprised the parenteral administration of quinine and, from the 1950s, chloroquine. When they became available, renal replacement therapies for adult patients with acute renal failure could also save lives [2].

Soon after Laveran’s discovery of the causative parasite, the pathological processes underlying severe malaria were elucidated by the great Italian malariologists Marchiafava and Bignami. They considered, correctly, that the sequestration of parasitized erythrocytes in the microvasculature, causing microcirculatory dysfunction, was the key pathological event in “malignant tertian” (severe falciparum) malaria [3, 4]. Beginning in the 1960s, coincident with the emergence of immunology as a discipline, and continuing to this day, various novel theories of severe malaria pathogenesis were proposed. These were often derived from observations in a murine “model” of cerebral malaria, which was fundamentally different to the human infection [2, 5]. These new theories spawned a long succession of putative adjuvant therapies for severe malaria. Unfortunately, none of these therapies worked, and several were harmful [2, 4, 5].

## 1985 WHO meeting

Before 1985, there was no standard definition of severe malaria. Cerebral malaria was defined as unrousable coma (no localizing response to a painful stimulus). After publication of the Glasgow Coma Scale (GCS) in 1976 [6], this level of coma became a GCS less than 11. In 1985 an “informal meeting” was convened by the Malaria Action Programme of the World Health Organization (WHO). It was held in the Institute for Medical Research in Kuala Lumpur where, decades before, Field and colleagues had conducted seminal studies on the diagnosis, pathology and prognosis of severe malaria. The WHO meeting had the objective of reviewing available information on severe falciparum malaria, standardizing the definition, and advising on management [7]. The resulting document, which derived heavily from studies in Thailand conducted in the previous five years [8], provided a definition of severe malaria which is broadly similar to that used today, but with the following exceptions.

- hyperparasitaemia was defined as > 5% parasitaemia (today this is 10%)
- after a convulsion, coma had to persist for 6 h (now 30 min),
- severe anaemia was defined as a haematocrit < 20% (now < 15%),
- jaundice (total bilirubin > 50 µmol/L) alone was a criterion (today this requires a parasite density > 100,000/uL as well),
- ‘fluid, electrolyte or acid–base disturbances requiring intravenous therapy’ was a criterion (today more specific criteria have been instituted: either a venous plasma lactate > 5 mmol/L, arterial pH < 7.25, or a plasma bicarbonate < 15 mmol/L is required).
- Hyperpyrexia (> 39 °C), vomiting of oral treatment and haemoglobinuria were also included – none of which today are considered defining criteria.

These definitions and descriptions have been generally referred to and referenced as “WHO definitions” although each successive version of the severe malaria review contains a disclaimer that the contents are the opinions of experts, and not those of the WHO itself.

## 1988 WHO meeting

In 1988 a second informal WHO meeting was held to update the recommendations and to incorporate recent observations in African children with cerebral malaria [9]. For the definition of severe malaria, hyperparasitaemia, jaundice, and hyperpyrexia were “dropped”, the haematocrit criterion was reduced to 15% and, after some debate, a requirement for a concomitant parasitaemia of > 10,000/µL was added to the severe anaemia criterion. Acidaemia or acidosis were defined as above, and repeated generalized convulsions (more than two observed within 24 h despite cooling) was added as a criterion. In this second meeting, the readily evaluated Blantyre Coma scale [10] was endorsed as the method to assess the level of consciousness in children.

## 1995 WHO meeting

The third WHO meeting was held in Geneva in December 1995 to incorporate further experience from clinical research in African children [10–12]. This meeting resulted in a broader, more inclusive and pragmatic, definition of severe malaria in children centred around prostration and respiratory distress (acidotic breathing) [13].

The hyperparasitaemia threshold was changed to 4% in low transmission settings, and to 20% in high transmission settings. The newly added “prostrate” criterion was very broad. It included many children with acute malaria who had no other signs of severity. This substantially expanded definition of severe malaria therefore encompassed a larger proportion of all children with acute malaria (and so it had a lower case-specific mortality). The new inclusive definition ensured a high proportion of at-risk children would be managed appropriately (i.e. it had high diagnostic sensitivity), but it had low specificity in identifying potentially fatal infections. In clinical research use of the broader inclusion criteria obviously resulted in overall “better outcomes” as more children with a good prognosis were included within the broader definition of severe malaria. Recognizing the disparities with the earlier criteria some investigators continued with the stricter (i.e. more specific) earlier severe malaria criteria [9] in their clinical research studies (Table 1).

**Table 1:** “Pragmatic” inclusive simple definition of childhood severe malaria [13]

Children at immediately increased risk of dying who require parenteral antimalarial drugs and supportive therapy (a) Prostrated children (prostration is the inability to sit upright in a child normally able to do so, or to drink in the case of children too young to sit) Three subgroups of increasing severity should be distinguished (i) Prostrate but fully conscious (ii) Prostrate with impaired consciousness but not in deep coma (iii) Coma (the inability to localize a painful stimulus) (b) Respiratory distress (acidotic breathing) (i) Mild-sustained nasal flaring and/or mild intercostal indrawing (recession) (ii) Severe-the presence of either marked indrawing (recession) of the bony structure of the lower chest wall or deep (acidotic) breathing	

## 2013 WHO meeting

The most recent WHO meeting on severe malaria was convened in 2013, again in Geneva [2]. By 2013, large prospective series of patients with severe malaria had been studied in Asia and Africa. These studies provided a much larger evidence base than for previous meetings. Many of the data came from randomized controlled trials [14–21]. The key therapeutic advance was the replacement of quinine by artesunate, which had been shown to reduce mortality by between one fifth and one third in very large randomized controlled trials [18, 19]. The definitions of severe malaria, and components of the definitions, could now be associated with mortalities [22–26] (which were falling globally as artemisinin combination treatments were rolled out and parenteral artesunate was replacing quinine as first line parenteral treatment) [2, 27]. The 2013 “WHO” meeting recognized both the requirements of a definition for practitioners, for whom sensitivity in recognizing potentially severe malaria and thus inclusiveness takes priority, with the contrasting needs of epidemiology and research studies where specificity is more important. The most recent research definition is shown in Table 2 [2].

**Table 2:** Strict research definition of severe malaria [2]

Impaired consciousness: A Glasgow Coma Score < 11 in adults or a Blantyre Coma Score < 3 in children Acidosis: A base deficit of > 8 meq/L or, if unavailable, a plasma bicarbonate of < 15 mmol/L or venous plasma lactate > 5 mmol/L. Severe acidosis manifests clinically as respiratory distress – rapid, deep and laboured breathing Hypoglycaemia: Blood or plasma glucose < 2.2 mmol/L (< 40 mg/dl) Severe malarial anaemia: A haemoglobin concentration < 5 g/dL or a haematocrit of < 15% in children < 12 years of age (< 7 g/dL and < 20%, respectively, in adults) together with a parasite count > 10,000/µL Renal impairment (acute kidney injury): Plasma or serum creatinine > 265 µmol/L (3 mg/dL) or blood urea > 20 mmol/L Jaundice: Plasma or serum bilirubin > 50 µmol/L (3 mg/dL) together with a parasite count > 100,000/µL Pulmonary oedema: Radiologically confirmed, or oxygen saturation < 92% on room air with a respiratory rate > 30/min, often with chest indrawing and crepitations on auscultation Significant bleeding: Including recurrent or prolonged bleeding from nose gums or venepuncture sites; haematemesis or melaena Shock: Compensated shock is defined as capillary refill ≥ 3 s or temperature gradient on the leg (mid to proximal limb), but no hypotension. Decompensated shock is defined as a systolic blood pressure < 70 mm Hg in children or < 80 mm Hg in adults with evidence of impaired perfusion (cool peripheries or prolonged capillary refill) Hyperparasitaemia: *P. falciparum* parasitaemia > 10%	

Prostration was not included in the ‘research” definition, convulsions were “dropped”, the acidosis criterion was refined, the jaundice criterion was reintroduced with a parasite density > 100,000/µL, and the hyperparasitaemia criterion was changed (again!). In addition, severe *Plasmodium vivax* and *Plasmodium knowlesi* infections were reviewed specifically, and slightly modified definitions for severe malaria with these infections were proposed [2].

## The meaning of severe malaria

Strictly speaking, severe malaria is malaria with an increased risk of death at the time of assessment compared to everyone else in that community with malaria illness. How much higher this risk should be (i.e. the lower threshold for the increase in mortality) has not been agreed upon. Mortality varies substantially as it depends on the infection, the host, the circumstances and the treatment. For the same admission severity, outcomes in well-equipped intensive care units (ICUs) with well-trained staff are better than in peripheral health centres. However, tertiary ICUs often receive the very sickest patients, often after long delays in referral – with consequently high mortalities. A frail and debilitated patient may die from a malaria infection that would be regarded as mild in a younger and fitter person. The high mortality of imported malaria (both *P. falciparum* and *P. vivax*) in elderly travellers, and of malariatherapy (all species) in neurosyphilis, testifies to the lethal potential of acute malaria illness, whichever the infecting parasite species, in frail or debilitated persons [28, 29]. In contrast, most people with malaria illness in endemic areas are either children or young adults without underlying conditions (although in Southern Africa HIV prevalence is high, and the untreated coinfection predisposes to severe malaria [30]). Acknowledging that this is an oxymoron, the “uncomplicated falciparum malaria” mortality of orally treated patients ranges from 1 in 10,000 to 1 in 1,000 if effective anti-malarial drugs are being used. Many factors affect this risk. Severe malaria usually has a mortality well over 5%, and therefore represents a > 50 fold increase in the risk of death. In general, as with many infections, mortality in malaria is proportional to the total number of infecting organisms (biomass) in the body. In non-immune adults mortality increases steeply as peripheral blood parasite densities rise over 100,000/µL [31]. This corresponds approximately to total parasite numbers within the blood of over 10^12^. If severe malaria was defined as clinical and laboratory measures which are associated with > 5% mortality, then the current thresholds would conform, except for the anaemia criterion (see below) which would require a threshold of 3 g/dL rather than 5 g/dL.

## Malaria parasite densities

Malaria is traditionally diagnosed by microscopy examination of a peripheral blood smear. Unfortunately, this diagnostic skill is being lost in many places as microscopy is replaced by the more ‘convenient’, but less informative, rapid diagnostic tests. In malaria microscopy, the parasites are speciated and their numbers counted. The result is reported either as the number of parasitized erythrocytes in a stained thin smear or, in a thick film, as the number of parasites seen in a fixed volume or while counting a certain number of white blood cells (usually 200 or 500). The old semi-quantitative ‘cross’ system, in which density is graded from + to +  +  +  + , is no longer recommended. The thin film should be used for high parasite densities (> 0.2% parasitaemia).

**Fig. 1:**
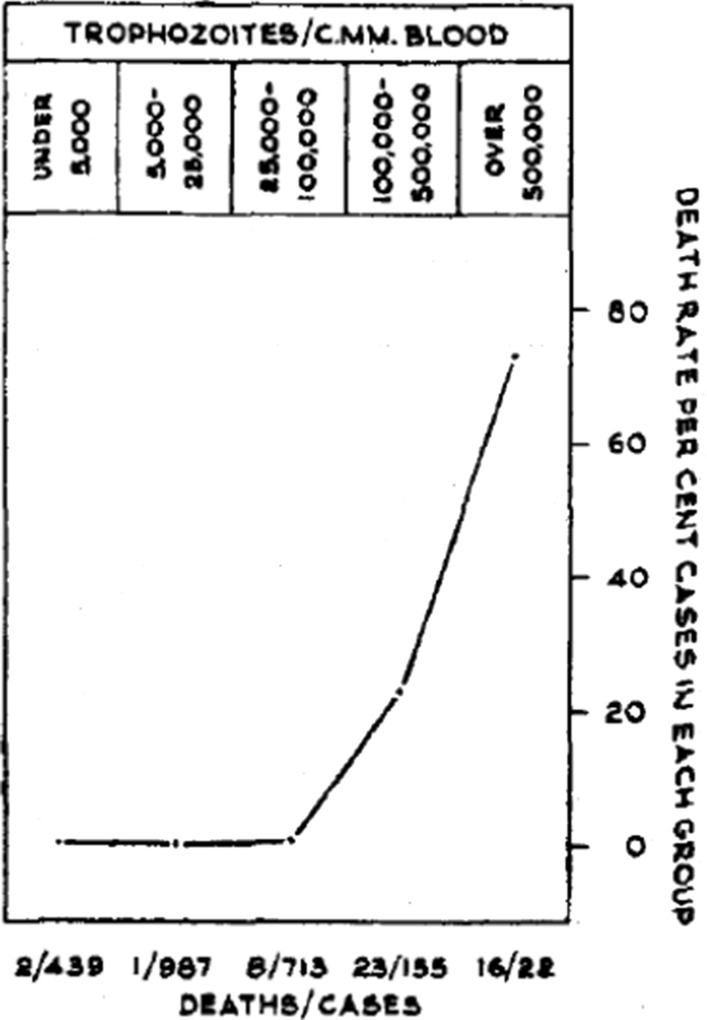
Relationship between peripheral blood parasite density and outcome in patients with acute falciparum malaria studied by Field and colleagues in Kuala Lumpur over 70 years ago [31]

In falciparum malaria the parasite count can be misleading. This is because after approximately 12 to 16 h (depending on core temperature) of intraerythrocytic parasite growth (i.e. one quarter to one third of the asexual life cycle) *Plasmodium falciparum* infected erythrocytes begin to stick (“cytoadhere”) to vascular endothelium. By 20 h the majority have cytoadhered. This “sequestration” is the fundamental pathological process in falciparum malaria [2, 3]. It occurs in all *P. falciparum* infections, although the tissue distribution of sequestration varies between patients. As a result, the parasite densities measured in blood films (reflecting circulating parasites only) variably underestimate the total malaria parasite biomass [32–34]. Nevertheless, the mortality of falciparum malaria is still proportional approximately to the peripheral blood parasite density. Among several factors, the relationship between peripheral blood parasite density and mortality depends on the prevailing intensity of transmission and thus the levels of “immunity” or “premunition”. Field showed in Kuala Lumpur (a generally low transmission area from the 1930s to the 1950s) that the mortality of falciparum malaria in adults with little or no immunity increased markedly when parasite densities rose above 100,000/µL [31] (Fig. 1). There is, therefore, a non-linear relationship between mortality and parasite densities. In a low transmission setting on the Thailand-Myanmar border, where the *P. falciparum* entomological inoculation rate was approximately 0.5/year, the mortality of children with > 4% *P. falciparum* parasitaemia (circa 200,000/µL) was 3% [35, 36]. In that location a 3% mortality was thirty times higher than the mortality in patients with lower parasite densities, but it was five times lower than in patients who fulfilled the strict WHO definition of severe falciparum malaria [9]. As the predominant stage of parasite development determines the proportion of the parasite biomass that circulates, some patients with severe falciparum malaria have relatively low parasite densities because most of the malaria parasites are sequestered [32–34]. Others may have low parasite densities because they have already received anti-malarial drugs before assessment. On the other hand, a synchronous infection may have recently undergone schizogony and merozoite release resulting in a high parasite density with a predominance of young ring stage parasites. In this latter case most of the parasites in the body are circulating, and relatively few are still sequestered. Provided the patient receives an artemisinin derivative the prognosis is good. In children in areas of higher transmission, *P. falciparum* peripheral blood parasite densities over 200,000/uL may be tolerated with relatively few symptoms. Thus, the prognostic value of parasitaemia depends on the epidemiological setting and, overall, it is poor.

## Factors associated with mortality

The three main clinical presentations of severe malaria in children are coma, metabolic acidosis (usually manifest by an acidotic or “Kussmaul’s” breathing pattern, and commonly termed “respiratory distress”) and anaemia [2, 10–13, 22–26]. None of these are specific for malaria. These clinical presentations are major manifestations in adults too, although severe anaemia is less common. In contrast many adult patients present with acute kidney injury often accompanied by jaundice [37]. As noted earlier, there is no agreed threshold mortality threshold to define severe malaria. Among the different syndromes included in the current definition, the lowest case specific mortality is associated with malarial anaemia which can be below 1% [38]. This is still higher (by a factor of 10–100) than in uncomplicated malaria, but it is substantially lower than the mortalities associated with coma, severe metabolic acidosis, pulmonary oedema or acute renal failure (8–50%) [2, 26]. The low mortality of severe anaemia with malaria is explained by the low sequestered parasite biomass and the inclusion, within the definition of severe malaria, of children with chronic anaemia (often as a result of repeated malaria attacks) and either incidental parasitaemia or a concomitant, otherwise uncomplicated, malaria illness. This is a very common presentation in high transmission settings where it is usually the main reason for blood transfusion in young children. The current “WHO” severe anaemia criterion requires an accompanying parasite density of 10,000/µL [2]. Densities in this range are often found in asymptomatic children, so may be incidental to the anaemia rather than causal. Even if causal the anaemia may result from a chronic process in which the parasite numbers are in a quasi-steady state, controlled by the immune response, and are very unlikely to increase further. If the parasite density requirement in the criterion for “severe anaemia” was raised it would be more specific for acute malaria but, even at higher densities, acute case specific mortalities do not rise above 5% until admission haemoglobin concentrations fall below 3 g/dL. However, it is still very important to recognize children admitted to hospital with severe malaria anaemia as a high risk group. These anaemic children have a high post-discharge mortality [39–41]). Furthermore they may not recover fully from their anaemia for 2–3 months after discharge. Thus, the overall mortality associated with severe malaria anaemia is significantly greater than appreciated from the acute admission [39, 40].

## The clinical syndromes

### Neurological dysfunction

**Fig. 2:**
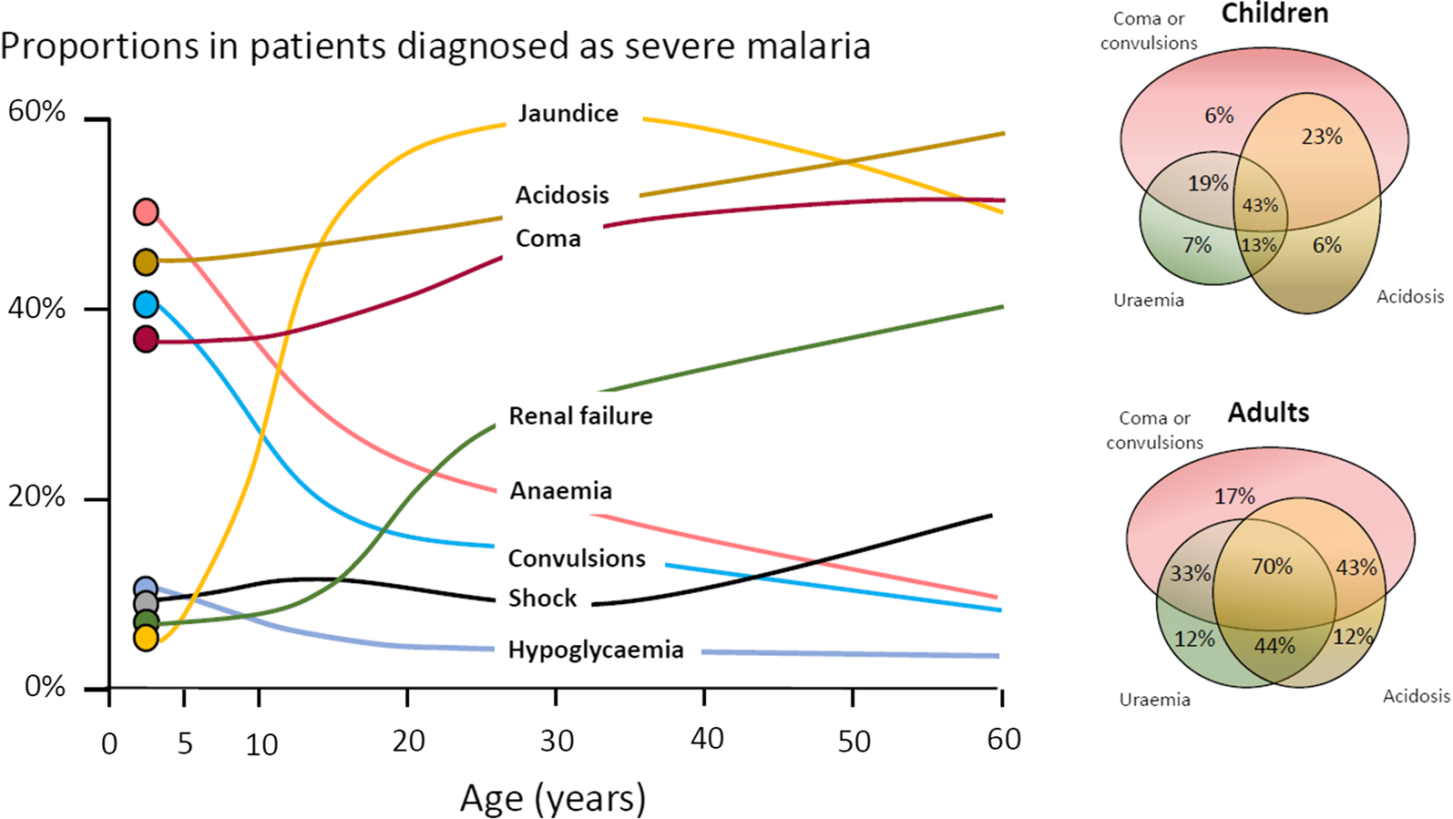
Overlap of clinical syndromes and mortalities in adults and children with severe falciparum malaria. These proportions are derived from prospective studies in SouthEast Asia and Africa of adults and children with severe falciparum malaria conducted or coordinated by the Mahidol Oxford Research Unit over the past 40 years [26]

**Fig. 3:**
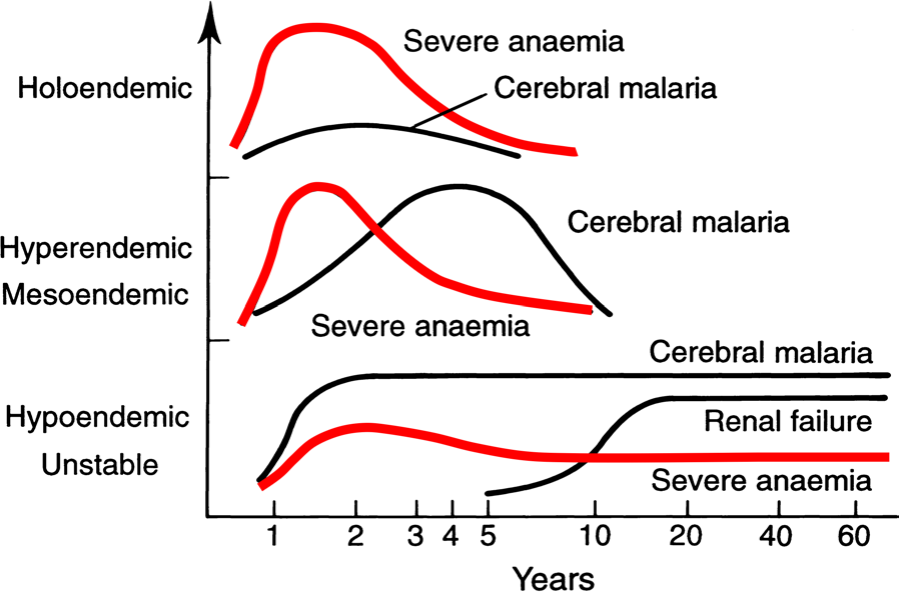
Approximate age relationships for the major clinical manifestations of severe falciparum malaria in relation to the intensity of transmission [53]. Holoendemic in this illustration approximates to a sustained entomological inoculation rate > 10 per year or a parasite rate (prevalence) in children of 0.5, and hypoendemic refers to an average entomological inoculation rate ≤ 1 year

The most characteristic syndrome of severe falciparum malaria is unrousable coma or cerebral malaria [2, 42]. This diffuse, symmetrical, reversible encephalopathy may occur at any age (Fig. 2). The main differential diagnoses are bacterial meningoencephalitis, viral encephalitis and, in some areas, toxic encephalopathy. Cerebral malaria occurs typically in people with little or no immunity, so it is seldom seen in residents of areas of high stable malaria transmission where severe anaemia in the first years of life predominates as the manifestation of severe malaria (Fig. 3). The outcome of cerebral malaria depends on access to treatment and intensive care, and the degree of associated vital organ dysfunction. ‘Pure’ cerebral malaria (i.e. without other vital organ dysfunction) has approximately half the mortality of patients with coma and other organ dysfunction i.e. renal impairment, pulmonary oedema, jaundice, metabolic acidosis, or hypoglycaemia. Overall, the treated mortality of cerebral malaria in the “quinine era” was approximately 20% in adults and 12–15% in children. These mortalities have been reduced by about one third by parenteral artesunate treatment [17–20]. Falciparum malaria is specifically associated with convulsions, even in otherwise uncomplicated infections. The seizures are usually generalized, and they may herald the onset of coma. Although most children make a full recovery, cerebral malaria in children is associated with significant neurodevelopmental sequelae; stroke, cognitive impairment and an increased risk of epilepsy [42]. It is very important to distinguish the causal relationship between convulsions in malaria and cerebral malaria and later cognitive impairment and epilepsy, from pre-morbid conditions which may present, sometimes for the first time, as neurological dysfunction in acute malaria (and thus be misdiagnosed as cerebral malaria). Otherwise, the adverse impact of cerebral malaria on long-term neurological outcomes will be overestimated. The specificity of the diagnosis of cerebral malaria is improved by clinical and laboratory examination (see below). For example, demonstration of malaria retinopathy is highly specific for cerebral malaria as the cause of coma [44]. Severe anaemia has also been associated with neurocognitive deficits [45]. There is no evidence that severe malaria causes permanent damage to other vital organs.

## Acidosis, kidney injury

Metabolic acidosis is a grave sign in both adults and children with severe malaria, [2, 24, 47–49] (Fig. 2), unless it results from very severe anaemia only, where the prognosis is better [38]. Lactate (reflecting lactic acid) accumulation is an important component of the malaria acidosis. Other organic acids, mainly of gut origin, are also significant contributors [46, 50]. Lactic acidosis is often accompanied by hypoglycaemia reflecting anaerobic glycolysis and impaired hepatic gluconeogenesis [47–49]. Impaired renal function is an important manifestation of severity in younger children, but acute kidney injury (AKI) requiring renal replacement therapies is almost confined to older children and adults [2, 37, 51] (Figs. 2, 3). The fulminant form of AKI, often associated with multiple vital organ dysfunction, is associated with a poor prognosis. In contrast the sub-acute presentation, in which plasma or serum creatinine rises steadily as the patient otherwise recovers, has a good prognosis. A period of renal replacement therapy (preferably haemofiltration or haemodialysis [52]) may be required, but there is always full recovery of renal function in survivors. The ‘hepatorenal’ combination of jaundice and renal failure became a more common presentation of severe malaria relative to cerebral malaria in Southeast Asia over the past four decades -the prognosis is worse than with AKI alone. Renal dysfunction in malaria can be misattributed in much the same way that neurological dysfunction following malaria can be overdiagnosed. In many tropical regions chronic kidney disease is common, particularly in older adults, and renal impairment may become evident for the first time during hospitalization for malaria. This may be causally attributed to malaria by mistake, and so a diagnosis of malaria nephropathy is made incorrectly. Concomitant anaemia and acidosis may also be ascribed incorrectly to malaria rather than chronic renal disease. In these misattributed cases, renal imaging, if available, often reveals small kidneys, or nephrolithiasis and hydronephrosis, and there may be biochemical or radiological evidence of metabolic bone disease.

## Severe anaemia

**Fig. 4:**
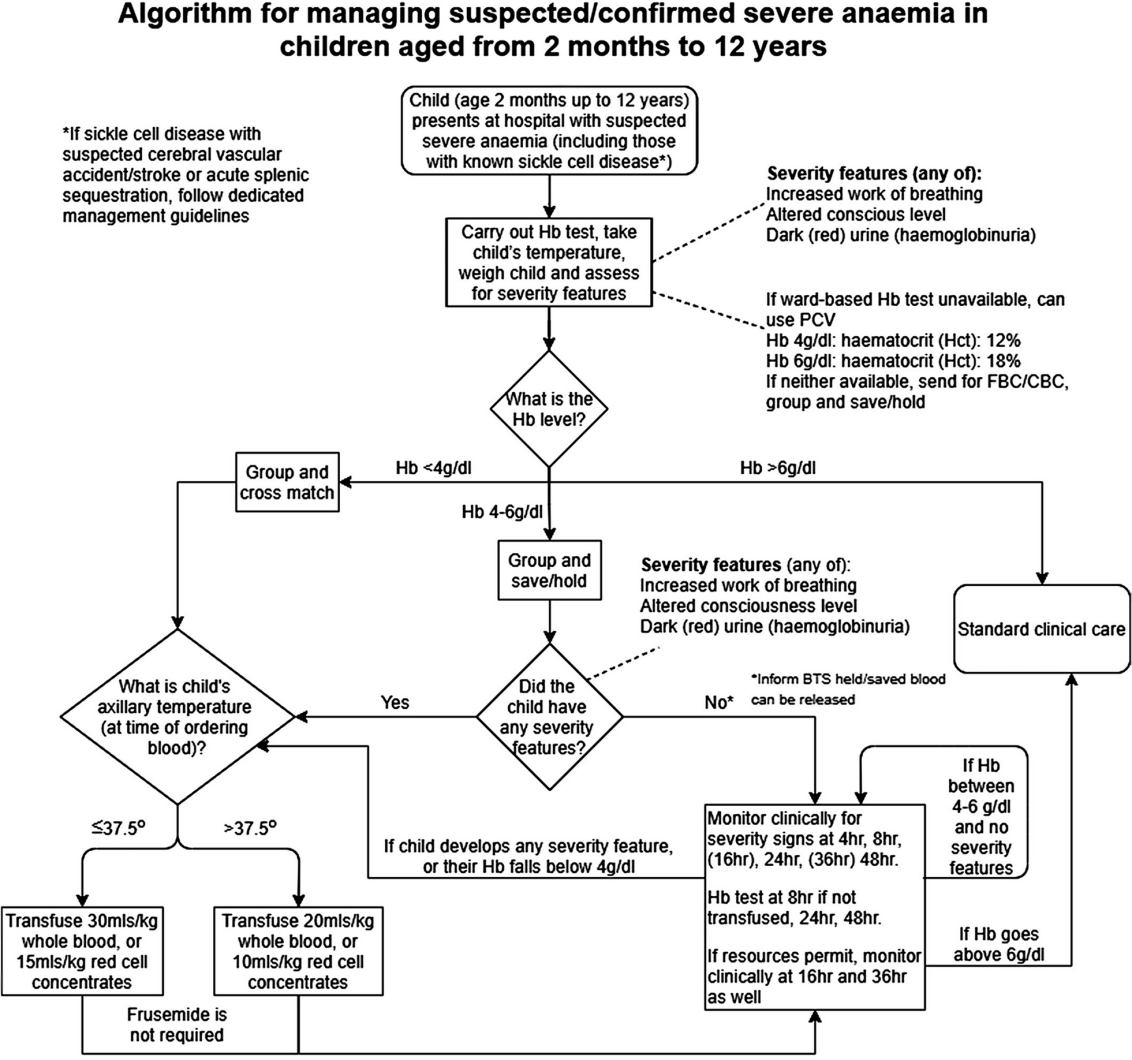
Proposed algorithm for managing suspected/confirmed severe anaemia in African children aged from 2 months to 12 years [58]

The definitions of anaemia in malaria vary widely [53]. The most common classification—used in higher malaria transmission settings- is based on haemoglobin concentrations. In patients with acute malaria haemoglobin (Hb) concentrations between 8 g/dL and 11 g/dL are considered as mild anaemia, Hb between 5 g/dl and 8 g/dL is considered moderate, and Hb < 5 g/dL is defined as severe anaemia [53]. Unfortunately, despite their simplicity, the point of care measurements of haemoglobin concentrations, which are necessary to ensure appropriate use of blood transfusions, are often unavailable [54]. In sub-Saharan Africa the Hb ≤ 5 g/dL threshold is used widely as an indication for blood transfusion in children with malaria (whereas Hb ≤ 4 g/dL is often used for other causes of anaemia) (Fig. 4). The recent finding, in a large randomized trial, that children with fever (> 37.5 °C) were harmed by higher blood transfusion volumes (30 mL/kg versus 20 mL/kg) whereas children without fever benefited [55–57], has forced a reconsideration of blood transfusion guidelines for African children with severe anaemia [58] (Fig. 4). In low transmission settings an Hb ≤ 7 g/dL has been used as a transfusion indicator [2]. There is no evidence to support this threshold. Anaemia is the main severe manifestation of malaria in areas of high transmission, where it is largely confined to young children [59] (Fig. 2). Severe anaemia, as a criterion of severe malaria, encompasses a spectrum of aetiologies with several different, but often overlapping, pathological processes which are still not well understood [53]. At one end of the disease spectrum is an acute illness in patients with high parasite biomass infections and rapid destruction of parasitized and unparasitized red cells. The unparasitized cells comprise the majority of erythrocytes lost. Haemolysis is sometimes sufficient to result in haemoglobinuria (blackwater fever). However, malaria is not the only cause of blackwater fever, which, after over 120 years of investigation, still remains a puzzle [60–64]. Massive haemolysis may occur in any epidemiological setting. At the other end of the disease spectrum, in settings of high transmission or poor access to treatment, are patients (usually young children) with chronic anaemia and incidental parasitaemia. Repeated or untreated malaria infections resulting in shortened erythrocyte survival and protracted dyserythropoeisis are important contributors to this chronic, or acute on chronic, syndrome [52].

Improved malaria control reduces the frequency of malaria infections and thus the prevalence of severe anaemia [59, 65]. As in chronic inflammatory conditions, malaria is associated with iron deficiency [66]. Other common causes of anaemia in malaria endemic regions are nutritional deficiencies, hookworm, bacterial infections and haemoglobinopathies. Bacterial infections are also associated with acute anaemia presentations [67]. At presentation to hospital the short-term prognosis of severe anaemia is relatively good as the anaemia is mainly chronic and partially compensated (by the right shifted oxygen dissociation curve). If blood transfusion can be given promptly then the acute mortality is low but, in higher malaria transmission settings, hospitalization for severe anaemia identifies children who are at increased risk of subsequent death. Approximately 5% will die within 6 months. Post-discharge anti-malarial chemoprophylaxis provides temporary protection, which suggests that recurrent malaria is a major contributor to this high mortality [40, 41]. The prognosis of children hospitalized with severe anaemia is much better than for the other severe manifestations of falciparum malaria but, because of the longer-term impact, and because it is so common in high transmission settings, the adverse impact at a population level is substantial [59]. Deaths from malaria overall are positively correlated with transmission intensity [59], and the direct or indirect consequences of severe anaemia are major contributors to this relationship.

## Other complications

Pulmonary oedema (ARDS) carries a very high mortality in falciparum malaria- even with positive pressure ventilation. It often occurs after the other severe manifestations have become evident. Pulmonary oedema results from increased pulmonary capillary permeability. Pulmonary oedema may also occur in vivax malaria, where the prognosis is much better [2]. Liver dysfunction is usual in severe malaria [68] although liver failure, as in viral or toxic hepatic injury, never occurs [2]. Profound thrombocytopenia is associated with an increased mortality in severe malaria, but it is not an independent risk factor and, contrary to some reports, it is not regarded as a criterion of severe malaria [69]. Although thrombocytopenia is usual in all malarias and coagulation indices are often abnormal in severe illness, significant bleeding (if present, usually from the stomach) and clinically significant coagulopathy are unusual in severe malaria. Overall, the probability of death from severe falciparum malaria depends on the extent and degree of vital organ dysfunction and the access to appropriate treatment [2, 70]. Secondary bacterial infection is a potentially lethal complication, particularly in African children. Approximately 6% of children diagnosed with severe malaria have concomitant bacteraemia [71]. In adults the incidence is much lower (1%) [72]. Misdiagnosis (see below) is common [73], as it is difficult to differentiate between severe malaria with concomitant bacteraemia and a primary bacterial infection with incidental parasitaemia [74, 75].

## Pathophysiology of severe falciparum malaria

**Fig. 5:**
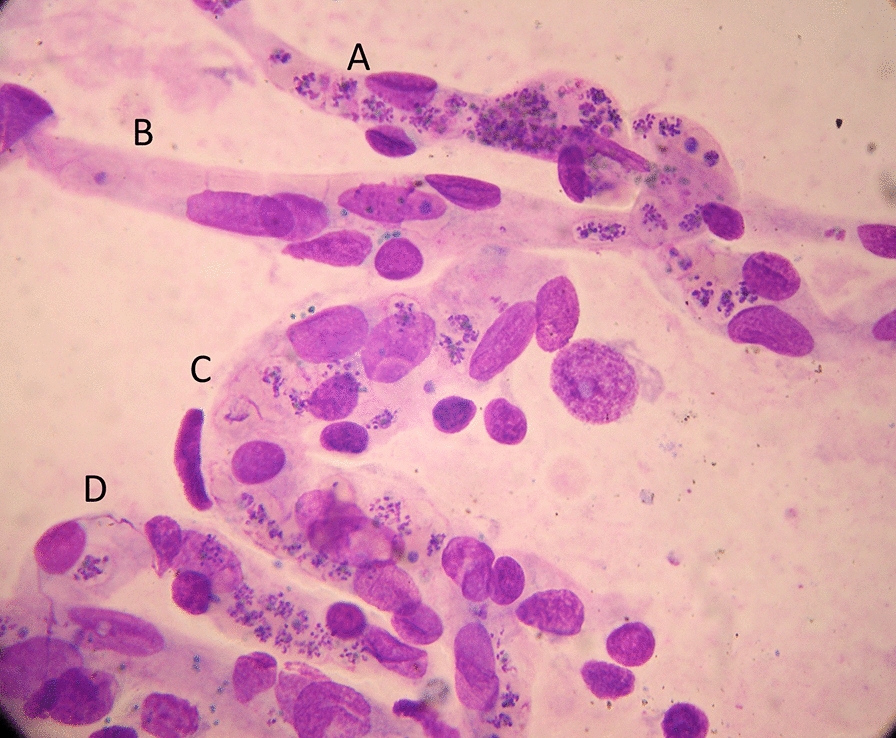
Brain smear from fatal cerebral malaria. The vessels (**A**, **C** and **D**) are packed with red cells containing *P. falciparum* schizonts (many of which are disrupted) and malaria pigment (haemozoin). Vessel segment **B**, by contrast, contains mainly unparasitized erythrocytes

Similar to some primate malaria parasites (*P. fragile, P. coatneyi*), but unlike the other human malaria parasites, *P. falciparum* causes the infected erythrocyte to cytoadhere to vascular endothelium after the first third of the asexual blood cycle [2]. Severe falciparum malaria results from the extensive sequestration of erythrocytes containing these mature parasite forms in the microvasculature of vital organs [2, 3, 76, 77] (Fig. 5). The microvascular obstruction by highly metabolically active cells, consequent cellular dysfunction, and the liberation of large quantities of bioactive haem are considered the main pathological processes in severe falciparum malaria [70, 76–79]. There are secondary consequences on vascular function, permeability, tone and on cellular transport. Thus, vital organ dysfunction depends on the extent and the location of parasitized erythrocyte sequestration. The extent of sequestration is heterogeneous, even at a microvascular level [80]. Magnetic resonance cerebral imaging in paediatric cerebral malaria shows a variety of different patterns. The brain is usually swollen, with restricted diffusion and variable evidence of oedema [81]. Isolated restricted white matter diffusion is associated with a better prognosis, while oedema is associated with a worse prognosis and an increased risk of sequelae [82, 83]. The sequestered static red blood cells occupy space and cause cerebral engorgement [3, 4] which contributes to raised intracranial pressure. Cytoadherent parasitised erythrocytes are not the only contributors to disease severity. Very high parasitaemias caused by non-sequestering malaria parasites cause severe malaria across the animal kingdom, and the simian parasite *P. knowlesi* is potentially lethal in humans – but these parasites do not cause cerebral malaria [2, 84]. At very high parasite densities, erythrocyte dysfunction contributes to aggregation and impaired microcirculatory flow and oxygen delivery without cytoadherence. The precise causes of acute kidney injury and acute pulmonary oedema in severe malaria are unclear. Despite extensive research and much speculation over many years, there is little evidence for a primary immunopathological process in severe malaria, or for a final common pathological pathway with bacterial sepsis involving pro-inflammatory cytokine release. As described earlier, the pathobiology of severe malaria has been rich ground for hypothesis and speculation, often fueled by observations in a murine model, which is readily studied in the laboratory but has very little similarity to the human disease [5]. Observations in the murine ‘model’ have led to a long list of putative adjuvant interventions -all of which have proved either ineffective or harmful. This emphasizes the importance of distinguishing causal pathological processes in malaria from their consequences. From a clinical and operational perspective, it is essential to distinguish causal processes in severe malaria [70] from those processes in other severe infections with which severe malaria is very often confused (notably bacterial infections). The implications of misdiagnosis on operational disease management and pathobiology understanding are discussed below.

## The diagnosis of severe falciparum malaria

Severe malaria is a medical emergency. Appropriate immediate management is life-saving. An initial brief clinical examination assessing vital signs, peripheral perfusion, respiratory pattern, anaemia, jaundice and level of consciousness, and confirming the absence of rash should be followed rapidly by a blood smear or RDT confirmation [2]. In a low transmission setting, or with imported malaria, the diagnosis is straightforward. The results of a thin blood film or RDT can be available within minutes of taking a blood sample. Treatment should not be delayed if the blood results take longer than this. Microscopy examination of thin and thick blood smears provides both diagnostic and prognostic information; the parasite count, the parasite stage of development and the presence of neutrophil ingested pigment all have prognostic value and are readily assessed [2, 32, 33, 85–87]. If the parasitaemia is high, the thin film assessment can take less than one minute. The RDT does not provide this quantitative prognostic information. In addition, the *Pf*HRP2 based RDTs can remain positive for days or weeks following a previous infection [88]. On the other hand, RDTs are useful in excluding a mixed *P. falciparum* infection in a patient with a blood slide diagnosis of vivax, malariae or ovale malaria [89], and they provide a diagnosis in patients who have received treatment with artemisinins several days previously and who are still severely ill (but have cleared their parasitaemia). This is common in adults presenting with acute kidney injury, which may take days or weeks to recover fully. In a low transmission setting, finding malaria parasites in the peripheral blood (by microscopy or RDT) is highly specific for malaria as the cause of illness. PCR diagnosis and speciation has proved very valuable in epidemiological studies, but PCR has no role in the acute diagnosis of severe malaria in endemic areas. It is too slow to be reported and it is too sensitive. PCR detects a higher proportion of people with previously asymptomatic (i.e. incidental) parasitaemia and therefore results in even more misdiagnosis of severe malaria.

At higher levels of transmission, the diagnosis of malaria as the cause of the presenting illness is much more difficult. The prevalence of microscopy or RDT detectable parasitaemia in apparently healthy individuals increases with transmission intensity, so the possibility of ascribing malaria incorrectly as the cause of illness rises too [90]. In sub-Saharan Africa a high proportion of apparently healthy children have detectable malaria parasitaemia. So how can severe illness caused by malaria parasites be distinguished from severe illness caused by something else with coincident parasitaemia? Good clinical examination is important but diagnostic uncertainty often persists. Other sites and sources of infection should be sought. In unconscious patients a lumbar puncture should be performed to exclude bacterial meningoencephalitis. Sequestration can be seen in-vivo by skilled indirect ophthalmoscopy along with other changes termed “malaria retinopathy” which have high specificity for cerebral malaria [44, 92–94]. The buccal or rectal microcirculations can be visualized by direct orthogonal polarized light imaging [76, 95]. In fatal cases sequestration can be demonstrated in the capillaries and venules of the brain in a post-mortem needle biopsy [80, 96, 97] (Fig. 5). But none of these specialist techniques are available in most places where severe malaria is managed. However, most hospitals and many health centres do have microscopes, and many centres now can perform full blood counts. Brief microscopy examination of a stained thin blood film provides valuable diagnostic and prognostic information [85–87]. The blood count is also informative (see below). Point of care blood glucose and lactate measurement is very important, particularly in unconscious or obtunded patients.

## The immediate management of severe malaria

The outcomes of severe malaria and of severe sepsis are critically dependent on rapid access to health care and immediate treatment. Delays in giving artesunate and antibiotics are potentially lethal. Sadly, additional delays may still occur after the patient has reached hospital. Any patient suspected of having severe malaria should be treated as such [2].

### Pre-referral

Severe malaria often presents initially far from the health centre or hospital. Referral for medical care can take hours, or sometimes days. At the community level, where giving parenteral drugs is not possible, pre-referral treatment of severe malaria with rectal artesunate reduces mortality by about 25% [98]. This community-based intervention has been very slow to be deployed, and now the WHO has recommended that it be stopped [99]. This recent WHO moratorium followed preliminary analysis of a large sequential observational study (“CARAMAL”) in Nigeria, Uganda and the Democratic Republic of the Congo [100]. Mortality reportedly increased after rectal artesunate was deployed, attributed to delays in the referral of severely ill children. However, there are serious concerns over the design of the study, potential major confounders, the accuracy of the diagnosis, and particularly—the causal interpretation of the results [101]. The CARAMAL study identified important problems with the referral of severely ill children, but it should not be used to evaluate the effectiveness of pre-referral rectal artesunate. The WHO moratorium appears to be a mistake. Rectal artesunate should be deployed to counter lethal delays in the referral of severe malaria. There are no pre-referral rectal antibiotic formulations unfortunately.

### Health centre or hospital

At the level of the health centre or hospital in an area of higher malaria transmission (i.e. most of sub-Saharan Africa), the difficulty in distinguishing malaria from sepsis in children means that both parenteral anti-malarials (i.e. artesunate 3 mg/kg stat for children < 20 kg and 2.4 mg/kg for larger patients) and broad-spectrum antibiotics should be given together as soon as the diagnosis is suspected [2, 73]. The most widely used empirical antibiotic treatment of severe sepsis is parenteral ceftriaxone. Administration of antibiotics should not be delayed. The drugs are very safe. Giving anti-malarials initially does no harm if the infection turns out to be bacterial or viral, and giving antibiotics does no harm if the infection is severe malaria only. Immediate administration of parenteral artesunate and broad-spectrum antibiotics to a child suspected of having severe malaria is *the single most important life-saving intervention*.

In low transmission settings where misdiagnosis is much less likely, it is reasonable in adults to treat only for severe malaria unless there is evidence for concomitant bacterial sepsis. However, antibiotics should be given to all adult patients with a very high parasitaemia (> 20%) [72], and should be given immediately if there is any unexplained clinical deterioration.

## The misdiagnosis of severe malaria

Misdiagnosis of severe malaria is common. Its impact is underestimated. Misdiagnosis can result in incorrect treatment [73] and it dilutes and distorts genetic, epidemiology, burden of disease, long term impact, pathophysiology and therapeutic studies. In areas of higher transmission (e.g. Sub-Saharan Africa, Oceania), children are often diagnosed as having severe malaria because the blood test is “positive” but, in fact, they have another infection (often bacterial sepsis) causing their severe illness [90]. As severe bacterial infections have a higher mortality than severe malaria, and require antibiotic treatment, it is essential that both are treated immediately.

**Fig. 6:**
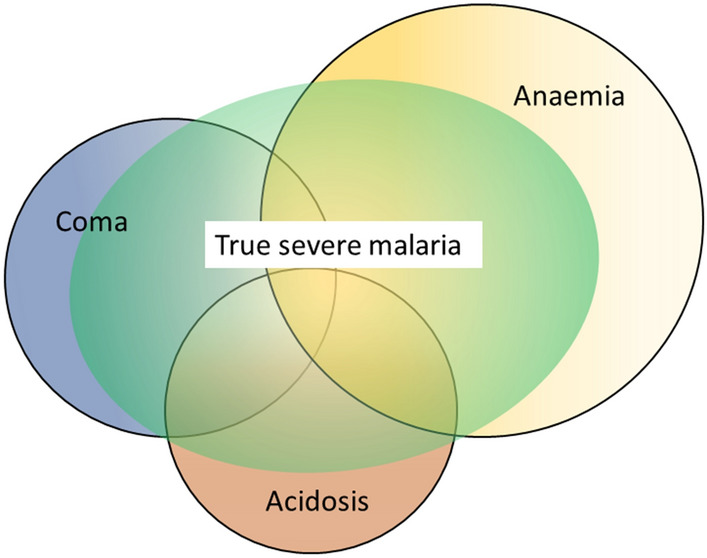
Misdiagnosis of severe falciparum malaria in African children -approximate relationships. [108, 109]

The relationship between malaria and bacterial infections is complex [71–75, 102–109]. Severe malaria predisposes to bacterial infections. In a large prospective series of Vietnamese adults with strictly defined severe falciparum malaria (in whom diagnostic specificity for severe malaria is very high), the overall incidence of concomitant septicaemia (identified by positive blood culture) was 1.1% [72]. Hyperparasitaemia was a risk factor for bacteraemia; in patients with > 20% parasitemia the prevalence of concomitant bacteremia was 5.2%, whereas it was eight times lower (0.65%) in patients with lower parasitaemias. Concomitant bacteraemia is much more frequent in African children diagnosed with severe malaria. Approximately 6% of children hospitalized with a diagnosis of severe falciparum malaria in Africa are also bacteraemic [71]. As blood cultures are insensitive (but more specific- at least for most organisms) in diagnosis, the true proportion is likely to be much higher. Recent probabilistic assessments based on platelet and white blood cell counts, and also a quantitative parasite biomass indicator (plasma P*f*HRP2) [110, 111] measured in large prospective studies of severe malaria in children, suggest that approximately one third of children diagnosed as having severe malaria in leading research centres actually had another condition (likely mainly sepsis) as the main cause of their illness [108, 109] (Fig. 6). These probabilistic assessments were validated by comparing the prevalences of sickle cell trait (HbAS), which provides strong protection against severe malaria, between the two groups. The prevalence of HbAS was substantially lower in children with ‘true’ severe malaria than it was in those with a different cause of severe illness. Even for the relatively specific syndrome diagnosed as cerebral malaria, a post-mortem examination study, conducted in a leading research centre in Malawi, revealed a different pathology in one quarter of cases [112].

**Fig. 7:**
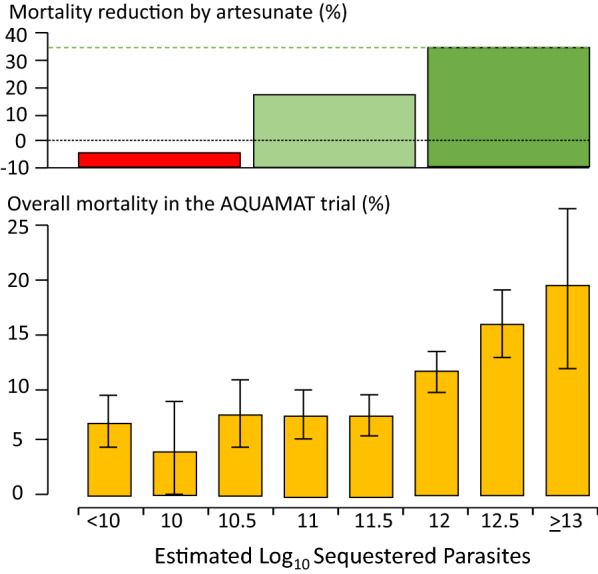
Relationship between estimated parasite biomass and mortality [4, 110] in the large randomized controlled trial which compared artesunate and quinine in African children with severe malaria (AQUAMAT) [19]. The upper panel divides the patients into tertiles by treatment effect (reduction in mortality by artesunate). The mortality reduction in the preceding randomized controlled trial (SEAQUAMAT) which compared artesunate and quinine in Southeast Asia (where the diagnosis of severe malaria is more specific) is shown for comparison [18] (upper green dashed line). There was no treatment benefit from artesunate in patients in the lowest tertile of parasite biomass (red), likely corresponding to patients with another cause of severe illness (probably sepsis) and incidental parasitaemia [108, 109]. The lower panel shows the corresponding relationship between mortality in the AQUAMAT study and the estimated total parasite numbers in the body derived from the admission plasma *Pf*HRP2 concentration [110]

The substantial overdiagnosis of severe malaria cannot be ignored in epidemiology, burden of disease, pathophysiology, genetic association and treatment studies. In the large evaluation of African children who had been admitted to leading research centres with a diagnosis of severe malaria (described above), mortality was higher in the likely misdiagnosed group, presumably because most had sepsis [108, 109]. This suggests that malaria attributable mortality in African children may have been overestimated. If it has indeed been overestimated then the benefits of the substantial investments in malaria control measures and the provision of effective drugs (i.e. ACTs) have been underestimated [113]. Progress in reducing the number of deaths from severe malaria may have been better than estimated currently. The high rates of misdiagnosis, even in expert research centres, should be also accomodated by those formulating treatment guidelines and policies for severe malaria. Prompt, or preferably pre-referral, antibiotics must be given together with artesunate. Misdiagnosis also probably explains the difference in mortality reduction with artesunate compared with quinine in adults and children in Asia (where diagnostic specificity is high) compared with children in Africa (where diagnostic specificity is lower) (22.5%) [5, 18, 19]. In Asia the mortality reduction was 35% compared with 22.5% in African children (Fig. 7).

Thus, it seems that some of the children with bacteraemia who are diagnosed as having severe malaria may genuinely have a high parasite biomass and extensive sequestration predisposing to bacterial sepsis—but the remainder have a primary bacterial infection and incidental or concomitant malaria. The interaction is complicated further as severe malarial anaemia predisposes to bacterial sepsis, and patients with uncomplicated malaria may have concomitant sepsis. At a population level, as malaria is controlled, the prevalence of sepsis declines (and so does the apparent protective benefit of HbAS against bacterial infections) pointing to the important contribution of malaria to bacterial sepsis, both concomitantly and sequentially [104]. It is very likely that the same problem of misdiagnosis occurs with *Plasmodium vivax.* In endemic areas low density chronic *P. vivax* parasitaemia is common, and so it is not unusual for severely ill patients to have incidental low-density infections, particularly if PCR is used for parasite detection.

## The consequences of severe malaria

Children who are admitted with severe malaria anaemia have a high mortality in the months following admission [39–41]. This can be reduced by giving effective antimalarial prophylaxis, which indicates that repeated malaria infection is associated with death. Seizures and coma are associated with neurological deficit in surviving children [42, 114]. The deficit is evident immediately following recovery in approximately 10% of children following cerebral malaria [115]. In two thirds of these cases the clinical picture is of stroke (suggesting a large cerebral vessel territory has been compromised). While many children recover fully, other deficits and behavioural and mental health problems often become apparent -particularly with detailed psychomotor and behavioural evaluation [42, 114, 116–118]. Epilepsy is increasingly recognized. These later onset epileptic, psychomotor and behavioural abnormalities may result from cerebral malaria, but they may also be pre-morbid conditions revealed by acute malaria [44].

## Implications for the assessment and treatment of patients diagnosed with severe falciparum malaria

Overall, the consensus definitions of severe malaria described generally as “WHO criteria” have worked well to identify patients at risk and to inform research studies. From a practical case management perspective, specificity in the diagnosis is not as important as recognition that severe malaria could be the cause of the severe illness, and thus starting life-saving treatment with artesunate as soon as possible [2]. A new simple to administer artesunate formulation is under development. In children with suspected severe malaria in higher transmission settings parenteral broad-spectrum antibiotics should also be given *immediately* in all cases. As delay in receiving artesunate is a major contributor to death, it is important that referral to a facility capable of managing the sick patient should be as rapid as possible. Pre-referral rectal artesunate should be given to all children with suspected severe malaria [2, 98]. The WHO moratorium [99] on rectal artesunate will hopefully soon be lifted [101]. Pre-referral antibiotic formulations should be developed.

For patients needing respiratory support, artificial ventilation has improved in recent years as the dangers of high inflation pressures have become evident [119]. Unfortunately, ventilators and trained staff are often unavailable in the areas where severe malaria is common. Otherwise, apart from the replacement of quinine by artesunate, the overall recommended management of severe malaria has changed relatively little over the past few decades. Aggressive fluid management (as in sepsis) [76, 120], high volume (30 mL/kg) blood transfusions (in febrile children)[56], mannitol to reduce brain swelling [121, 122], and unproven adjuvant therapies [5] have all proved harmful. Studies to optimize blood transfusion and fluid management are ongoing, but the general consensus is returning back to more cautious fluid management in severe malaria [7, 123]. Evidence to date does not support red cell concentrates over whole blood in immediate management [57]. The optimum prevention and treatment of convulsions still remains uncertain. In a large randomized trial, conducted in a centre without access to artificial ventilation, seizure prevention by full dose prophylactic phenobarbitone increased mortality because of respiratory depression [16]. In a small trial levetiracetam proved safer [124], and may well become the anticonvulsant of choice, as it is in other settings, although more evidence is needed. Fosphenytoin was ineffective [125]. Renal replacement should start early in adults, blood glucose should be tested frequently and hypoglycaemia treated promptly [2]. Studies are ongoing to determine if paracetamol could attenuate renal injury in severe malaria [51]. If broad spectrum antibiotics have not been started (e.g. in adults in low transmission settings) there should be a low threshold for giving them if the patient deteriorates [2] -particularly in hyperparasitaemic patients [72].

From a research or epidemiology perspective, the low specificity of the current definition of severe malaria in African children is a challenge (Fig. 6). It has diluted therapeutic evaluations and distorted pathophysiology interpretations and genetic association studies. Most of the techniques to improve the specificity of diagnosis (notably indirect ophthalmoscopy or other methods of visualizing the microcirculation, or measurement of parasite biomass indicators such as plasma *Pf*HRP2 (Fig. 7) or plasma *Pf*DNA concentrations) are not readily available [91–95, 110, 111, 126]—although simple dilution of a plasma sample and testing (by eye) with a *Pf*HRP2 RDT is not too difficult [127]. Importantly, the time-honoured peripheral thin blood smear does contain valuable information. Sadly, it is underused as a diagnostic and as a prognostic tool, and in many centres has been supplanted by the malaria rapid test, which, as currently used, does not provide prognostic information. In blood slides with parasitaemias over 0.5% the stage of parasite development can be easily and rapidly evaluated by microscopy. For any parasite density, finding > 50% tiny rings carries a relatively good prognosis whereas if > 20% parasites contain visible malaria pigment the prognosis is worse [85]. The proportion of neutrophils containing malaria pigment is also a very useful and readily assessed both for diagnosis and for prognostic assessment [86, 87]. Most health facilities have at least one microscope – but sadly it is often old, fungus infested and accompanied by dirty slides, waterlogged methanol and outdated unfiltered stains. Malaria microscopy is well established but it is not well supported, and it is not prioritized in current malaria control funding. Hospital and health centres managing severe malaria should support good microscopy as an essential diagnostic and prognostic measure. Blood counts are valuable too. The haemoglobin concentration or haematocrit guides blood transfusion. The differential white count provides diagnostic information. Although severe malaria may be accompanied by leukocytosis, finding a high neutrophil count (often with toxic granules) together with lymphopenia points to bacterial sepsis. Thrombocytopenia is usual in severe malaria, but not in sepsis. In the recent large probabilistic assessments of severe malaria in African children, the combination of a platelet count of ≤ 150,000/μl and a plasma *Pf*HRP2 concentration of ≥ 1000 ng/ml had an estimated sensitivity of 74% and specificity of 93% in identifying true severe falciparum malaria [109] (Table 3). Future studies of severe malaria should always include differential blood counts, platelet counts and, preferably, a parasite biomass indicator. The anaemia criterion to define severe malaria should be reviewed.

**Table 3:** Probabilistic evaluation of patients admitted to large research centres with an initial diagnosis of severe falciparum malaria [108, 109]

Measure	Values in African children with probable misdiagnosed severe falciparum malaria compared with true severe malaria	Comment
Parasite counts	Lower	Thin films should be examined. Thick film counts are harder to evaluate and less reliable
Haemoglobin	Higher	
White blood cell counts	Higher	Neutrophilia and lymphopenia suggest bacterial infection, although moderate neutrophilia may also occur in very severe malaria
Platelet counts	Higher	Thrombocytopenia is usual in malaria. Platelet counts over 200,000/µL are unusual in severe malaria
Plasma *Pf*HRP2 levels	Lower	Usually unavailable as a laboratory measure, but simple adaptation of current RDTs may substitute
Malaria pigment containing neutrophils	Less	Very easy to assess in the tail of a thin film. > 5% indicates worse prognosis. Usually negative in sepsis (unless concomitant hyperparasitaemia)
Positive blood cultures	More	Bacteraemia may occur in ‘true’ severe malaria, but misdiagnosis is more likely
Mortality	Higher	The treated mortality of septicaemia is higher than that of severe malaria

## Severe malaria caused by other malaria species

*Plasmodium knowlesi,* with its quotidian cycle, can sometimes cause fulminant infections in humans [84, 128, 129]. It does not sequester markedly so the parasite count is a good guide to biomass. *P. knowlesi* infections do not cause coma (cerebral malaria) but they can cause the other potentially lethal manifestations of severe malaria. Morphologically the younger *P. knowlesi* parasites resemble *P. falciparum*, whereas the older forms are often mistaken for *Plasmodium malariae*. Indeed any *P. malariae* parasitaemia over 1% should be regarded as *P. knowlesi* until proved otherwise. Uncomplicated *P. vivax* infections in a non-immune subject are often worse than uncomplicated *P. falciparum* malaria infections, causing high fever, weakness, malaise and sometimes rigors and prostration. Some of these vivax malaria illnesses warrant hospital admission. In the past 20 years there has been a marked increase in the number of reports of “severe” vivax malaria, mainly from India [130–132]. In some of the reports, the basis for the classification has been thrombocytopenia, which is not generally regarded as a criterion for severe malaria. Some patients hospitalized with *P. vivax* malaria die, particularly if they are old or debilitated [133, 134]. *P. vivax* may sometimes cause acute pulmonary oedema-although the prognosis is better than in severe falciparum malaria [133, 135]. But severe vivax malaria is overdiagnosed for the same reasons that severe falciparum malaria is overdiagnosed. Incidental parasitaemias are found in patients with severe anaemia or vital organ dysfunction and a causal relationship is inferred. In low transmission settings (i.e. most *P. vivax* endemic areas) *Plasmodium vivax* can cause severe illness, but the proportion of symptomatic cases which develop life-threatening illness is substantially less than for *P. falciparum* infections. However recurrent infections with *P. vivax* in areas of high transmission, such as the island of New Guinea, are associated with severe anaemia and substantial mortality both in the acute phase and over the longer term [136–138]. Further large and detailed cohort studies of hospitalized *P. vivax* infections would help clarify the prognostic associations and risk factors. But overall, the mortality of acute *P. vivax* infections is substantially lower than that of *P. falciparum* infections.

## Conclusions

The apparent lack of progress in reducing the global death toll from malaria despite substantial investment suggests that we should reexamine the evidence, and review the current strategies to prevent and treat severe malaria [113]. The mortality of this common but frequently misdiagnosed syndrome can and should be reduced. Severe malaria deserves more attention.

## Data Availability

Review—individual trial data from trials conducted by MORU can be requested from the MORU data access committee.
